# Opening Study on the Development of a New Biosensor for Metal Toxicity Based on *Pseudomonas fluorescens* Pyoverdine

**DOI:** 10.3390/bios3040385

**Published:** 2013-12-10

**Authors:** Alessandro Chiadò, Luca Varani, Francesca Bosco, Luca Marmo

**Affiliations:** 1 Department of Applied Science and Technology, Politecnico di Torino, Corso Duca degli Abruzzi, 24, 10129, Torino, Italy; E-Mails: francesca.bosco@polito.it (F.B.); luca.marmo@polito.it (L.M.); 2 Institute for Research in Biomedicine (IRB), Via Vincenzo Vela 6, 6500, Bellinzona (CH), Switzerland; E-Mail: luca.varani@irb.usi.ch

**Keywords:** heavy metals, pyoverdine, environmental monitoring, minimum inhibitory concentration, optical detection

## Abstract

To date, different kinds of biosensing elements have been used effectively for environmental monitoring. Microbial cells seem to be well-suited for this task: they are cheap, adaptable to variable field conditions and give a measurable response to a broad number of chemicals. Among different pollutants, heavy metals are still a major problem for the environment. A reasonable starting point for the selection of a biorecognition element to develop a biosensor for metals could be that of a microorganism that exhibits good mechanisms to cope with metals. Pseudomonads are characterized by the secretion of siderophores (e.g., pyoverdine), low-molecular weight compounds that chelate Fe^3+^ during iron starvation. Pyoverdine is easily detected by colorimetric assay, and it is suitable for simple online measurements. In this work, in order to evaluate pyoverdine as a biorecognition element for metal detection, the influence of metal ions (Fe^3+^, Cu^2+^, Zn^2+^), but also of temperature, pH and nutrients, on microbial growth and pyoverdine regulation has been studied in *P. fluorescens.* Each of these variables has been shown to influence the synthesis of siderophore: for instance, the lower the temperature, the higher the production of pyoverdine. Moreover, the concentration of pyoverdine produced in the presence of metals has been compared with the maximum allowable concentrations indicated in international regulations (e.g., 98/83/EC), and a correlation that could be useful to build a colorimetric biosensor has been observed.

## 1. Introduction

Microorganisms are well-adapted to their own ecosystem, and the degree of their adaptability can be evaluated through different measurable parameters (e.g., biomass growth, metabolism by-products, protein expression). When an environmental variable changes, the microorganisms react by altering their biochemical behavior. One of these mechanisms can be used to build environmental biosensors: these are widely applicable devices that combine a biorecognition element with a signal transducer to obtain a new, on-site, analytical tool [1]. Among a variety of application fields, biosensors are still predominantly used in the medical and environmental fields, and the monitoring of contaminants improves the knowledge and management of risks posed by chemicals to human health and the environment [1]. Furthermore, a sensible element can be implemented in wireless networks, thus obtaining a so-called BEWS [2], or biological early warning system, that continuously controls the overall quality of ecosystems. 

Metals are natural constituents of the environment: they can be found in minerals or as organometallic compounds [3]. Some of them, such as sodium, potassium, copper, zinc, iron, calcium, magnesium, cobalt and manganese are essential for life; these elements only become toxic when present in high concentrations. However, other elements that are not biologically relevant (e.g., cadmium, mercury and lead) are toxic, even at very low concentrations, because of their bioaccumulation properties [3]. Industrial pollution and acid rain increase the bioavailability and dispersion of metals, which are regarded as priority pollutants by worldwide institutions and organizations [4]. A proper sensible element that could be applied in metal biosensing could be selected from among the microorganisms that are able to survive and react to harmful concentrations of metals: the biological reaction could be exploited as a response in a biosensor. Many different microorganism (e.g., lichens, algae, fungi [3,5]) have been used for biomonitoring. For instance, lichens are really good biomarkers of pollution given by a broad spectrum of chemicals, although they are not really suitable for the development of a BEWS, as proven by results from a New Zealand case study [6]. 

Although iron is essential for the growth and development of almost all living organisms, as it acts as an enzymatic cofactor, promoting electron transfer or participating in oxygen metabolism, its availability for microbial assimilation in the environment is extremely limiting, because it is mostly insoluble [7]. In highly aerobic conditions, the predominant form of iron is ferric, which is soluble in water at about 10^−18^ M [8]. This concentration is too low to sustain the growth of microorganisms, which usually need concentrations close to 10^−6^ M [7]. Different strategies have been adopted by microorganisms to cope with this limited bioavailability. A widely used mechanism is to secrete siderophores [3,9]: these compounds are low-molecular-weight chelating agents (200–2,000 Da), that show an extremely high affinity for Fe^3+^, a feature used for iron uptake via active transport systems through the cell membrane [10,11,12,13]. 

At the same time, bacteria and fungi show that metals other than iron stimulate siderophore production [3,10,14]. The aptitude of toxic metals to regulate siderophore production suggests that these compounds may also be relevant in microbial heavy metal resistance [10,15]. High concentrations of metals may interfere with microorganism siderophore-iron uptake pathways, and at the same time, heavy metal toxicity may be modulated by the concentration of siderophores [15]. As an example, the binding of heavy metals to siderophores considerably reduces their bioavailability and protects the microorganism by affecting the uptake process, as proposed for a Cd-resistant bacterium [16]. 

Pyoverdine is one of the main siderophores secreted by fluorescent *Pseudomonads* for iron uptake in combination with pyochelin [12,17]. Siderophores regulation in *Pseudomonads* has mostly been investigated in *P. aeruginosa* PAO1 (ATCC 15692), and it has been revealed that both siderophores have a protective role on heavy metal toxicity [10,15]. Siderophores are produced under iron-limiting conditions, but they are also able to chelate other metals with lower affinity [10]. For complete insight into the pyoverdine regulation mechanism, reference can be made to various reviews [11,12,13,15]. 

Siderophores have great therapeutic (e.g., drug delivery), as well as analytical prospects [18]. However, during the last few years, only a few pyoverdine-based biosensors have been studied and developed, most of which are molecule-based [19,20,21], while only one is a whole-cell bioassay [22]. Furthermore, all of these biosensors were intended for the determination of ferric ion in medical or pharmaceutical applications. 

This paper describes the first results relative to the development of a whole-cell biosensor for environmental monitoring of different metals in water. The pyoverdine of *P. fluorescens* was evaluated taking into account that factors other than iron limitation influence the production of siderophores in *Pseudomonads* [23], e.g., temperature, pH and the carbon source. The influence of these parameters and of different metal ions on growth and pyoverdine regulation was investigated. Moreover, the pyoverdine production in the presence of metals was compared with the environmental quality standards established in international regulations (98/83/EC; WHO guidelines for drinking water quality). 

## 2. Experimental Section

*P. fluorescens* DSMZ 50090 (ATCC 13525) was streaked on a DSM1 agar plate [24] and grown overnight at 20 °C (preculture phase). The plate was then re-suspended with 10 mL of saline solution (0.9% NaCl) and used as an inoculum. The cultures were prepared using a succinic acid medium (M78) [23] or substituting the carbon source with glucose (4 g/L). In order to evaluate the effect of different initial pH, the ratio of the salts in the phosphate buffer (K_2_HPO_4_/KH_2_PO_4_) was modified in the succinic acid medium. Cultures were set up in baffled Erlenmeyer flasks (500 mL), in BOD bottles (500 mL) or in 96-well plate (Corning Incorporated-3799), at 15–30 °C and 130 rpm, in microaerobic conditions, and 1–10% of inoculum with an optical density at 620 nm (OD_620_) of 0.8–1.0 RU was added. 

pH was recorded with a Crison 2001 pH meter. Cell growth was monitored by measuring the OD_620_ (HP 8452A Diode Array Spectrophotometer or Biotek PowerWave 340 Microplate Reader). Pyoverdine was estimated by measuring the OD at 400 nm (OD_400_) of the culture supernatant obtained by means of centrifugation (Centrifuge 4217, AIC) (3,000 rpm, 20 °C, 10 min). Concentrations of succinic acid were determined by means of a high performance liquid chromatograph (HPLC) (Kontron Instrument) equipped with an ion exchange column (Hamilton HC-75 H, 305 × 7.8 mm), at 50 °C, using 5 mM H_2_SO_4_ as the mobile phase [25]. Each sample was filtered (0.22 μm, cellulose acetate, Sartorius Stedim Biotech GmbH) and analyzed on an HPLC using a refractive index and UV-Vis (λ = 210 nm) detectors. 

Minimum inhibitory concentrations (MICs) were determined on DMS1 agar plates, using a modified Kirby-Bauer method [24], and in liquid media, using 96-well plates [26]. As far as the modified Kirby-Bauer method is concerned, the inhibition halo area allowed the values of the MICs to be calculated for CuSO_4_, ZnSO_4_, FeCl_3_ and Fe_2_[SO_4_]_3_ [24]. Instead, DSM1 broth or M78 medium was used to determine the MIC in 96-well plates and in Erlenmeyer flasks and was supplemented with increasing concentrations of each metal (up to 10 mM). The cultures were incubated for at least 48 h, and growth was monitored by measuring the OD_620_, as stated above. In order to study the growth and pyoverdine production in the presence of metals, cultures were set up in a baffled Erlenmeyer flask or in 96-well plates, as stated above; otherwise, the culture media was supplemented with different Fe^3+^, Cu^2+^ or Zn^2+^ concentrations (up to 6.25 mM). The concentrations for these tests always referred to the metal ion. 

All experiments were performed with at least two replicates. All the chemicals used were purchased from Sigma-Aldrich (Milan, Italy). 

## 3. Results and Discussion

### 3.1. Influence of Physical-Chemical Parameters on Growth and Siderophore Production

The influence of physical-chemical parameters is one of the first steps in the assessment of a new biological sensing element, particularly for the development of a portable device [2]. 

**Figure 1:**
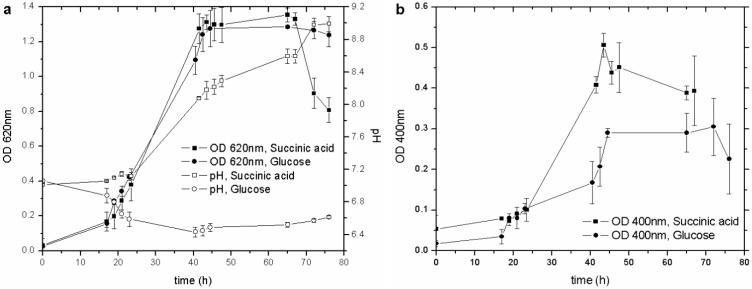
(**a**) Influence of different C sources on biomass growth and pH behavior; (**b**) influence of different C sources on pyoverdine production. OD, optical density.

The pyoverdine production in *P. fluorescens* seems to be influenced by different carbon sources [23]. Cultures with glucose and succinic acid, by far the most commonly used to culture this strain, were set up to establish the best C source for the pyoverdine synthesis. Both types of culture showed similar growth behavior (OD_620_) (Figure 1(a)), with the siderophore content increasing along with the biomass concentration, from the early exponential growth phase until the culture entered the stationary one. Nevertheless, succinic acid produced more pyoverdine (nearly double) than glucose did (Figure 1(b)), even though the C/N ratio was unchanged (17.6 for glucose and 17.9 for succinic acid). 

A possible explanation for this result is that the pyoverdine molecule is composed of a succinate moiety [27]: this compound in the culture medium represents an advantage for the microorganism, as it saves energy for the cell metabolism and biosynthesis. In spite of the smaller amount of siderophore produced, the cultures carried out with glucose were less variable in terms of pH and OD_620_. In each test conducted with succinic acid, the OD_620_ fell when the pH was higher than 8.4, while the culture with glucose remained stable during the whole stationary phase, with a recorded pH of about 6.5. In other papers, it has been reported that *P. fluorescens* cultures grown in succinic acid reach high pH values, of about 8.0–8.8, during the stationary phase [4]. It is feasible that the depletion of succinic acid, buffered at pH 7.0, left an excess of OH^-^ in the M78 medium, with a resulting increase in pH and a detrimental effect on the viability of the microorganism. 

These outcomes highlight the direct influence of the carbon source on microbial growth and on siderophore production in *P. fluorescens*. One of the most relevant results is that the higher production of pyoverdine attained with succinic acid as the C source leads to the development of a biosensor with a wider dynamic range than the one achievable with glucose. Therefore, the C source in the culture media for the subsequent trials was succinic acid. 

**Figure 2:**
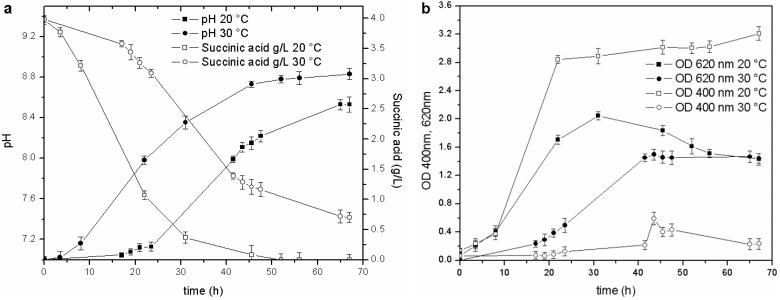
Cultures performed at 20 and 30 °C: (**a**) pH trends and C source concentration; (**b**) biomass growth and pyoverdine behavior.

Temperature was the second physical-chemical parameter that was evaluated. Tests were performed in BOD bottles (500 mL) and in baffled Erlenmeyer flasks (500 mL), over a 15–30 °C range, to assess the effect of temperature on growth and siderophore production, under different agitation conditions. Biomass growth and siderophore production were higher in the 15–20 °C range in both experimental devices. The pyoverdine content was three to ten times higher than in the cultures maintained at 25–30 °C. An example of the culture set-up in baffled Erlenmeyer flasks is reported in Figure 2. 

The stationary phase was reached in less than 20 h in the culture maintained at 20 °C (OD_620_ 2.0 RU), while the duration of the exponential phase at 30 °C was longer (40 h), and the maximum OD_620_ attained in the stationary phase was only 1.35 RU. Likewise, a faster consumption of the carbon source was observed at 20 °C than at 30 °C: the C source in the former culture was almost depleted at 45 h of fermentation (pH 8.7), while, the stationary phase in the culture maintained at 30 °C was reached at about 40 h (pH 8.2), and at the end of the test, when the pH value was 8.6, the succinic acid concentration was still 0.7 g/L. These results also prove that the decrease in the growth rate is probably related to a C source limitation (succinic acid below 0.5 g/L). 

The influence of temperature on pyoverdine production has been studied in detail in *P. aeruginosa*: this strain has an optimal temperature for pyoverdine production at 30 °C [28] and a higher optimal growth temperature, near 37 °C [29]. The results obtained with *P. fluorescens* grown at different temperatures also underline this behavior for this strain: the optimal growth temperature (20–25 °C, on OD_620_ and a cell dry weight basis) was higher than the optimal temperature for pyoverdine production (15–20 °C). The temperature was therefore controlled at 20 °C for the subsequent trials. 

**Figure 3:**
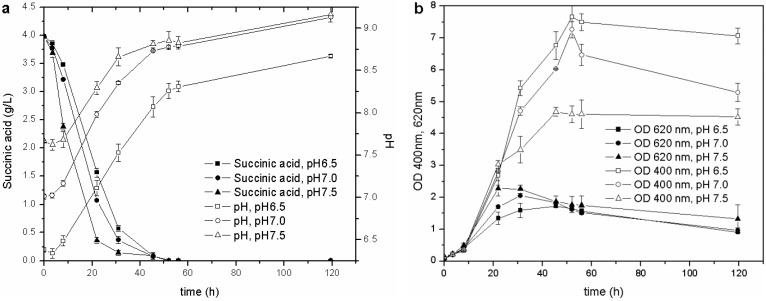
Cultures carried out at different initial pH: (**a**) pH and C source consumption; (**b**) growth and pyoverdine production.

Once the optimal carbon source and temperature for growth and siderophore production were established, the influence of the initial pH was evaluated. The biomass growth, pH values, C source consumption and pyoverdine biosynthesis of the cultures started from the M78 media buffered at different pH are reported in Figure 3. This test showed that the higher the initial pH, the faster the growth of the microorganism during the exponential phase and, consequently, the earlier the stationary phase is reached. The behavior of pH and carbon source consumption was related to the growth curve of the cultures. The stationary phase was achieved when the pH was about 8.0, and the succinic acid was almost depleted (concentration lower than 0.5 g/L) after 20, 30 and 40 h for pH 7.5, 7.0 and 6.5, respectively. This observation further confirms that the drop in OD_620_ recorded during the stationary phase is probably related only to a C source limitation (succinic acid concentration <0.5 g/L): the microorganism seems to grow faster at basic pH, as can be deduced from the carbon source consumptions and OD_620_ trends. 

During the exponential growth phase, the siderophore production (OD_400_) was very similar for the different cultures, but once the stationary phase was attained, the lower the starting pH, the higher the pyoverdine secreted by *P. fluorescens*. The influence of pH on the pyoverdine production is probably related to the optimal pH value of the culture. Pyoverdine is produced by the cells over time, and its concentration depends on the amount of living cells, but also on the period of time in which they grow. In the present experiments, the cultures, which started at different pH, reached the stationary growth phase at different stages over intervals of 10 h (Figure 3(b)), and the one that started at the lowest pH remained for a longer time in the optimal growth phase and pyoverdine production pH range than those cultures that started at a higher pH. This test has revealed that the initial pH value influences not only the microbial growth, but also the siderophore synthesis throughout the entire stationary phase. 

### 3.2. Determination of Minimum Inhibitory Concentration (MIC) of Fe^3+^, Cu^2+^ and Zn^2+^ on a Solid Medium

**Figure 4:**
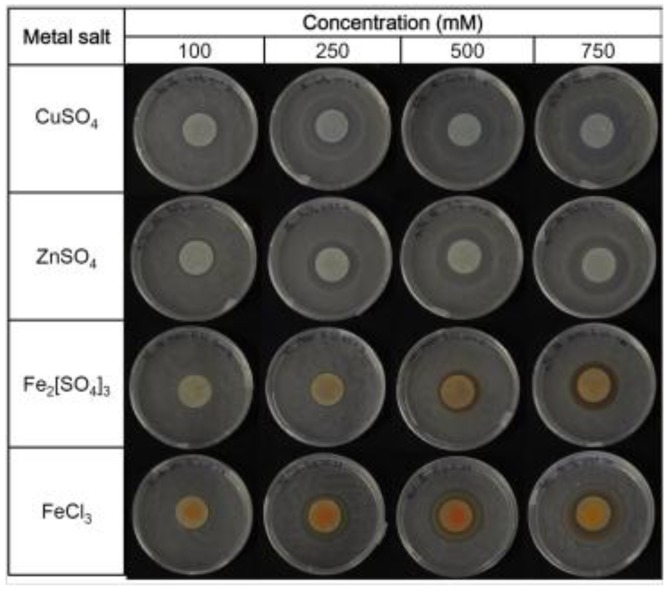
Minimum inhibitory concentrations (MICs) of Fe^3+^, Cu^2+^ and Zn^2+^ obtained on agar plates.

The interactions between the metals (Fe^3+^, Cu^2+^ and Zn^2+^) and *P. fluorescens* were then investigated applying a modified Kirby-Bauer test [24]. The first step was to determine the minimum inhibitory concentration (MIC), which is defined as the lowest metal concentration for which the growth is inhibited after overnight incubation [26] (Figure 4). The area of the inhibition halo measured applying different metal concentrations allowed the values of the MICs to be calculated for CuSO_4_, ZnSO_4_ and Fe_2_[SO_4_]_3_, and these resulted in 46.30, 54.40 and 74.11 mM, respectively. Two different salts were tested for Fe^3+^ to evaluate the counter-ion effect: SO_4_^2−^ was less toxic than Cl^−^ [24]. 

The values of MIC determined on the solid media were high compared to the MIC values established in the liquid cultures, and only the order of the metal sensitivity of the strain (Cu^2+ ^> Zn^2+^) agrees with the results reported by Poirier *et al*., for the high metal resistant *Pseudomonas* BA3d12 [30], or by Teitzel and Parsek [31]. It is reasonable to assume that mass transfer limitations, absorption and metal bioavailability affected the results obtained on the solid media more than those attained in liquid cultures. As a consequence, lower inhibitory concentrations are achieved in liquid cultures, as mentioned by other authors [32,33,34], and the only advantage offered by solid media during the MIC determination is that the bioavailability of the metals is similar to that of soil [33], where ions tend to be adsorbed to particles or complexed to organic compounds, such as organic acids (e.g., oxalic acid or humic acid) [3]. Although the values of MIC attained with the modified Kirby-Bauer test on the solid media highlighted that the biorecognition element was fully compatible with the concentrations of metals usually found in unpolluted freshwater (e.g., 98/83/EC, Table 2 in Subsection 3.4), it was clear that the interaction of the microorganism with metals should also be investigated in liquid cultures. 

### 3.3. Evaluation of the Influence on Growth and Siderophore Production of Fe^3+^, Cu^2+^ and Zn^2+^ in Erlenmeyer Flasks

Owing to the bioavailability limitations of the tests on solid media, the interaction of *P. fluorescens* with Fe^3+^, Cu^2+^ and Zn^2+^ was investigated in metal supplemented liquid cultures, carried out in well-mixed, baffled Erlenmeyer flasks. 

**Figure 5:**
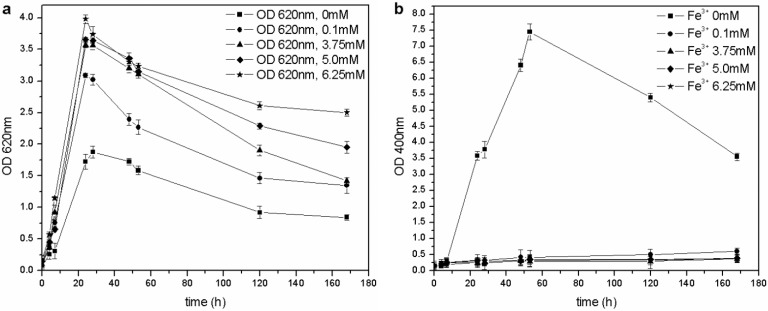
(**a**) Biomass growth in cultures supplemented with FeCl_3_. (**b**) Pyoverdine production (OD_400_) in cultures supplemented with FeCl_3_.

The microorganism was able to grow at each tested iron concentration, and no toxic effects were observed in the 0–6.25 mM range of Fe^3+^, unlike the results by Workentine *et al*. [35] (Table 1). Furthermore, when the concentration of ferric ion in solution was increased, faster growth and higher maximum OD_620_ values were recorded, compared to the control (0 mM FeCl_3_) (Figure 5(a)): the presence of a low concentration of metals could be an advantage during microbial fermentation and, in particular, for the growth of *P. fluorescens* [14,23]. Moreover, the pyoverdine production chiefly depends on the Fe^3+^: if the concentration is above a critical value (non-limiting iron or critical iron concentration for pyoverdine production, or CICP), the siderophore synthesis is repressed by the microorganism. During this test, siderophore was only produced in the control flask (0 mM Fe^3+^) and not by the other cultures (Figure 5(b)), confirming that the CICP is lower than 0.1 mM, in agreement with the results of other authors [23]. 

**Table 1:** Comparison between MICs obtained in liquid cultures (mM).

Metal	This work	Workentine *et al.* [35]	Poirier *et al.* [30]
**Fe^3+^**	>6.25	6.25	/
**Cu^2+^**	0.1–0.95	3.13	0.66
**Zn^2+^**	>2.0	1.56	0.95

**Figure 6:**
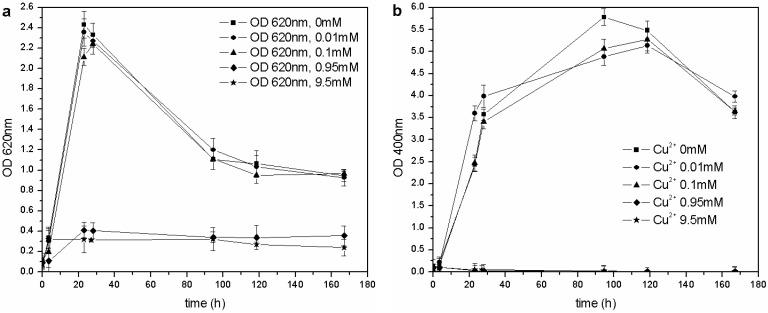
(**a**) Biomass growth in cultures supplemented with CuSO_4_. (**b**) Pyoverdine biosynthesis (OD_400_) in cultures supplemented with CuSO_4_.

**Figure 7:**
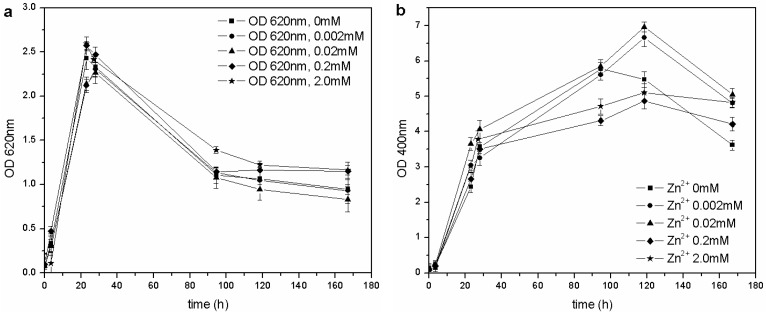
(**a**) Biomass growth in cultures supplemented with ZnSO_4_. (**b**) Pyoverdine production (OD_400_) in cultures supplemented with ZnSO_4_.

The same kind of experiment was carried out with CuSO_4_ and ZnSO_4_. As far as copper is concerned (Figure 6(a)), *P. fluorescens* was only able to grow in the 0–0.1 mM range of Cu^2+^, and each flask showed similar growth behavior (OD_620_ values), pH trends and carbon source consumption. The other cultures (0.95–9.5 mM of Cu^2+^) were inhibited completely, and no changes were in fact observed in the pH values and C concentrations. According to these results, the MIC for CuSO_4_ in liquid cultures is roughly three times less than that reported by Workentine *et al.* [35], but is similar to the one reported by Poirier *et al.* [30] for the strain, BA3d12 (Table 1). However, the determined MIC for ZnSO_4_ was higher than that reported by the previously mentioned authors: the microorganism was able to grow at each tested concentration of Zn^2+^, and no toxic effects were detected in the 0–2.0 mM range (Figure 7). 

The same concentration of pyoverdine was obtained in those cultures supplemented with Cu^2+^, in which the microorganism was able to grow (0–0.1 mM Cu^2+^). A similar result was obtained in cultures supplemented with Zn^2+^: the siderophore content was similar to that of the control flask (0 mM Zn^2+^), except for a greater concentration in cultures supplemented with 0.02 and 0.002 mM Zn^2+^ during the stationary phase of growth. As previously reported [14,28], it is known that 10–100 µM concentrations of Zn^2+^ can improve growth and pyoverdine production in the related *Pseudomonas aeruginosa*. 

These experiments carried out in Erlenmeyer flasks have proven that the MICs obtained in liquid cultures are lower than those attained on solid media. Moreover, higher (for Fe^3+^ and Zn^2+^) or lower (for Cu^2+^) concentrations inhibited the growth of the microorganism compared to the MICs determined by other authors [30,35] (Table 1). These discrepancies could be due to the effect of different culture conditions, such as the use of diverse experimental devices (96-well plates instead of flasks), higher or lower inoculum percentages and rich or minimal culture media. Owing to the large number of variables, and in order to use the same small-scale applied by other authors [31,35], the tests that were carried out in Erlenmeyer flasks were also performed in 96-well plates. 

### 3.4. Evaluation of the Influence on Growth and Siderophore Production of Fe^3+^, Cu^2+^ and Zn^2+^ in 96-Well Plates

The MICs in the 96-well plates were assessed by means of the serial dilution method [26], initially using two different culture media: a complex medium (DSM1) and a minimal one (M78). The obtained results clearly confirmed that microbial growth is influenced by the presence of the metals and also by the type of culture media: the MIC was higher for Cu^2+^ and Zn^2+^ in the complex medium (DSM1), while the MIC for Fe^3+^ was higher in the minimal medium (M78). Generally, the highest MIC values are obtained for a complex growth medium, as reported by other authors [31,32]: metal bioavailability is probably influenced because of a higher level of ion complexation by the medium components. This hypothesis is appropriate for copper and zinc, but not for iron: the concentration of Fe^3+^ required for microbial growth, which was not sufficient in the minimal medium [23], was probably re-established by supplementing the M78 medium. Instead, only a toxic effect was recorded for Cu^2+^ and Zn^2+^. 

Some solubility problems were encountered in these experimental tests: with the addition of a concentrated metal solution to both culture media, which were already rich in salts, the solubility product was reached easily, insoluble precipitates were formed and the nominal concentration of bioavailable metal was reduced. In order to confirm the precipitate production in the absence of biomass, a control test was conducted with both media, at different metal concentrations in non-inoculated 96-well plates, and the optical density was evaluated (OD_600_ and OD_400_). As soon as the solutions were mixed, a precipitate formed as the metal concentration increased, especially in the presence of chloride salts, and the solubility problems were much more evident in the presence of iron and copper. 

**Figure 8:**
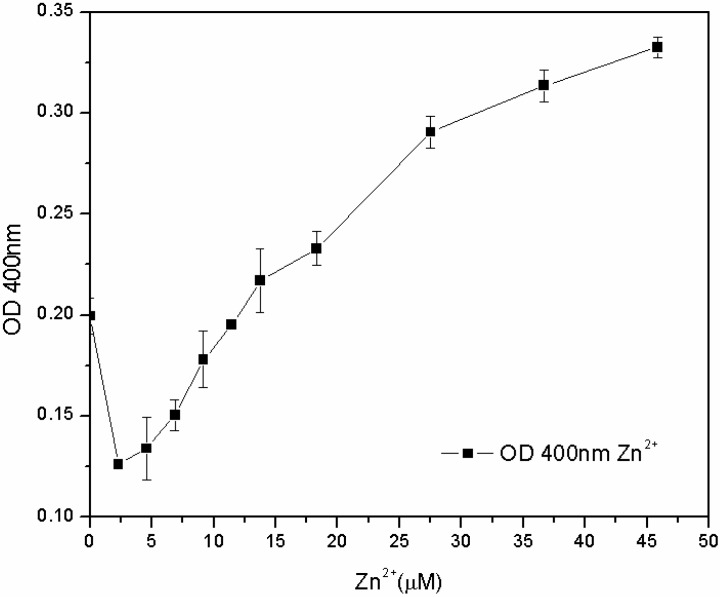
Pyoverdine (OD_400_) at 24 hours in 96-well plates cultures treated with low Zn^2+^ concentrations.

The subsequent tests, carried out in the M78 medium with a lower range of metal concentration and with different inoculum percentages, showed that the susceptibility of the microorganism for Fe^3+^ and Cu^2+^ depends not only on the concentration of metal, but also on the inoculum percentage: the lower the biomass in the inoculum, the lower the MIC. On the other hand, the microorganism grew for each zinc concentration, and after 24 h, the pyoverdine content was directly proportional to the concentration of Zn^2+^ in the 7.6–46 µM range (Figure 8). The reasons for this effect are not clear. One hypothesis is that the siderophore content secreted by the microorganism from zero to 7.6 μM of Zn^2+^ is sufficient to sustain growth, without any toxic effect. At 7.6 μM, Zn^2+^ became toxic to the microorganism, which increased its pyoverdine production as a protective mechanism. It is known that pyoverdine is effective in shielding the microbial cell of related *Pseudomonads* from Cu^2+^ and Zn^2+^ toxicity [10,15]. Unfortunately, in this work this effect was only observed for zinc and not for copper; further investigations are required to have a clearer picture of the interactions between *P. fluorescens* and these different metal ions. 

From these results, it is clear that the MICs of metals depend on several variables (e.g. the medium, the inoculum percentage), and this makes it difficult to compare data reported by different authors and obtained in diverse experimental conditions. For instance, regarding Cu^2+^, Teitzel and Parsek [31] found a MIC of 127 mg/L for *Pseudomonas aeruginosa*, Chen *et al.* [36] established 190 mg/L for *Pseudomonas putida*, whereas Tom-Petersen *et al.* [37] encountered 3 mg/L for *P. fluorescens*. These data highlight the heterogeneity of MIC, which is closely related to the tested strain and conditions. This complexity of iron uptake is further influenced by the simultaneous presence of different siderophores: pyochelin synthesis in *P. aeruginosa* is repressed by the same concentration that induces pyoverdine synthesis [14,38]. Teitzel *et al.* [39], through 2D-electrophoresis, verified that exposure to Cu^2+^ upregulates the genes involved in the synthesis of pyoverdine and downregulates those involved in the synthesis of pyochelin [10]. A similar experimental approach should be used to investigate this effect in *P. fluorescens* ATCC 13525. 

**Table 2:** Comparison between MICs, critical iron concentrations for pyoverdine (CICPs) and EU and WHO drinking water regulations (μM).

Metal	MIC	EU	WHO	CICP
**Fe^3+^**	1,500.0	3.6	5.4	3.6
**Cu^2+^**	100.0–500.0	31.5	31.5	25.0
**Zn^2+^**	46.0–500.0	/	46.0	/

These tests performed in 96-well plates have also been useful to investigate the CICP for Fe^3+^, Cu^2+^ and Zn^2+^ at different concentrations. By comparing the attained values with those indicated in different international regulations for drinking water (Table 2), it is possible to see that the MICs of Fe^3+^, Cu^2+^ and Zn^2+^ are always above the threshold specified in the EU drinking water directive (98/83/EC) and in the WHO guidelines for drinking water quality. At the same time, the concentration of metal ions required to inhibit pyoverdine production (CICP) is very close to the recommended thresholds for iron and copper. Instead, CICP was not determined for zinc in the tested concentration range (0–46 μM) and conditions. 

This last experiment has highlighted that *P. fluorescens* is well-suited to grow in the range of allowed concentrations of metals in water, and then, the correlation between the environmental quality standards and the CICP of Fe^3+^ and Cu^2+^ can be used to build a colorimetric biosensor, with the hopeful prospects of the *in situ* application of *P. fluorescens* pyoverdine. 

## 4. Conclusions

This introductory study has shown that pyoverdine production in *P. fluorescens* is influenced by the carbon source, temperature and the initial pH value. The microbial growth and pyoverdine production were higher when succinic acid (above 0.5 g/L) was used instead of glucose as the carbon source, and the temperature was controlled at 15–20 °C. As far as pH is concerned, the lower the starting value, the higher the pyoverdine secreted by the microorganism during the stationary phase. 

Once the influence of the physical-chemical parameters had been evaluated and the agreement with those values usually found in the aquatic environment had been assessed, the interaction between *P. fluorescens* and metals was studied considering solid and liquid media. The values of MICs obtained on the solid media were not comparable with the literature values, unlike those attained in Erlenmeyer flasks and in 96-well plates. 

Furthermore, these experiments have revealed that *P. fluorescens* metal susceptibility depends on the concentration of metal and also on the culture conditions (e.g., medium and inoculum percentage). 

The obtained MIC and CICP values were compared with the environmental quality standards: the MICs of Fe^3+^, Cu^2+^ and Zn^2+^ were always above the specified threshold, while the CICPs were very close to the recommended thresholds for iron and copper. These results have highlighted that this biorecognition element requires further investigation (e.g., with other metals and mixed differently), but the optimistic outcomes would seem to show that pyoverdine regulation could be useful for building a cheap biosensor that would be able to broadly detect toxic metals in the environment. 
